# Antibiotic Resistance Trends in Uropathogens during the COVID-19 Pandemic in Western Romania: A Cross-Sectional Study

**DOI:** 10.3390/antibiotics13060512

**Published:** 2024-05-30

**Authors:** Elena Hogea, Alexandra Cristina Muntean, Felix Bratosin, Iulia Georgiana Bogdan, Oana Plavitu, Adelina Fratutu, Cristian Oancea, Mihai Calin Bica, Delia Muntean, Ingrid Hrubaru, Zoran Laurentiu Popa, Adrian Cosmin Ilie

**Affiliations:** 1 Department XIV, Discipline of Microbiology, Victor Babes University of Medicine and Pharmacy, 300041 Timisoara, Romania; hogea.elena@umft.ro; 2 Clinical Hospital of Infectious Diseases and Pulmonology “Dr. Victor Babes”, 300310 Timisoara, Romania; dr.oanaplavitu@yahoo.com (O.P.); oancea@umft.ro (C.O.); 3 Emergency Clinical County Hospital “Pius Brinzeu”, 300723 Timisoara, Romania; ciocea.cristina@yahoo.com; 4 Methodological and Infectious Diseases Research Center, Department of Infectious Diseases, Victor Babes University of Medicine and Pharmacy, 300041 Timisoara, Romania; felix.bratosin@umft.ro (F.B.); iulia-georgiana.bogdan@umft.ro (I.G.B.); 5 Lupeni Municipal Hospital, 335600 Lupeni, Romania; adelina.ade2811@yahoo.com; 6 Center for Research and Innovation in Precision Medicine of Respiratory Diseases, Victor Babes University of Medicine and Pharmacy, 300041 Timisoara, Romania; 7 Discipline of Pulmonology, Victor Babes University of Medicine and Pharmacy, 300041 Timisoara, Romania; 8 Doctoral School, Victor Babes University of Medicine and Pharmacy, 300041 Timisoara, Romania; 9 Department of Microbiology, Victor Babes University of Medicine and Pharmacy, 300041 Timisoara, Romania; muntean.delia@umft.ro; 10 Department of Obstetrics and Gynecology, Victor Babes University of Medicine and Pharmacy, 300041 Timisoara, Romania; hrubaru.ingrid@umft.ro (I.H.); popa.zoran@umft.ro (Z.L.P.); 11 Department III Functional Sciences, Division of Public Health and Management, Victor Babes University of Medicine and Pharmacy, 300041 Timisoara, Romania; ilie.adrian@umft.ro

**Keywords:** antibiotic resistance, drug resistance, COVID-19, antibiotics, urinary tract infections

## Abstract

The emergence and spread of antimicrobial resistance have been significant global health challenges, exacerbated by the COVID-19 pandemic. As healthcare systems faced unprecedented pressures, the management of non-COVID conditions, including urinary tract infections (UTIs), also encountered obstacles due to changes in microbial flora and antibiotic usage patterns. This cross-sectional study aimed to characterize the antimicrobial resistance trends among bacterial uropathogens isolated from patients in the Western region of Romania, between January 2020 and December 2022. The objectives were to map the resistance patterns and observe the pandemic’s influence on antimicrobial resistance, particularly among enterobacterial Gram-negative species, to guide treatment and infection control strategies. From a total of 2472 urine samples collected during the study period, 378 positive samples were analyzed. This study found that *Escherichia coli* was the most commonly isolated uropathogen, making up 46.3% of the cases (n = 175), with *Klebsiella pneumoniae* at 20.6% (n = 78). There was a high resistance of *Klebsiella pneumoniae* to several antibiotics, while carbapenemase production increased to 52.5% and extended-spectrum beta-lactamase (ESBL) present in 24.3% of the strains. *Escherichia coli* showed high resistance rates to amoxicillin–clavulanic acid (from 45.4% in 2020 to 53.8% in 2022) and trimethoprim/sulfamethoxazole (from 27.5% in 2020 to 47.2% in 2022). The increasing trend of antimicrobial resistance noted during the pandemic, especially in Gram-negative enterobacterial species, highlights the urgent need for robust infection control measures and rational antibiotic use. This study underscores the critical importance of continuous surveillance to adapt antibiotic therapies effectively and prevent the further spread of resistance, thereby ensuring effective management of UTIs in the evolving healthcare landscape influenced by the pandemic.

## 1. Introduction

The onset of the coronavirus disease 2019 (COVID-19) pandemic marked a significant public health challenge globally. Declared a Public Health Emergency of International Concern by the World Health Organization on 30 January 2020, and subsequently upgraded to a pandemic on 11 March 2020, COVID-19 has rapidly affected populations worldwide [1,2]. It has led to over 750 million cases and more than 6.85 million fatalities globally. The pandemic has catalyzed profound disruptions in healthcare systems, notably impacting antibiotic prescription behaviors and increasing hospital-acquired infections, which in turn have exacerbated the spread of antibiotic-resistant bacteria [3,4,5,6,7,8,9]. These systemic disturbances have significantly influenced the management of common bacterial infections, such as urinary tract infections (UTIs).

Urinary tract infections represent a prevalent global health issue, with an annual incidence estimated at 150 to 250 million cases. These infections, which can range from mild cystitis to severe pyelonephritis, pose a significant burden across all demographics but particularly affect young women and the elderly [10,11,12,13,14,15,16,17,18,19,20,21,22,23]. The conventional diagnostic approach involves culturing the causative pathogens and conducting sensitivity tests, a process that can delay the initiation of appropriate treatment and increase the risk of severe outcomes, such as urosepsis.

The predominant pathogen in these infections is *Escherichia coli*, although research across Europe has also identified other Gram-negative bacteria such as *Klebsiella* spp., *Proteus* spp., and *Pseudomonas* spp., as significant causative agents. Furthermore, Gram-positive bacteria like *Enterococcus* spp. are implicated in around 20% of cases [24,25,26,27,28]. Current guidelines from the European Association of Urology (EAU) recommend tailoring antibiotic therapy to reflect local antimicrobial resistance patterns to ensure effective and evidence-based treatment decisions, thereby reducing the risk of complications and minimizing disease progression [29,30,31,32]. Therefore, the primary objective of this study is to assess the antibiotic susceptibility of Gram-negative bacteria isolated from patients with UTIs in the Western region of Romania during the COVID-19 pandemic. This analysis aims to elucidate the local antimicrobial resistance trends and compare them with global patterns, thereby contributing valuable insights into the evolving dynamics of antibiotic resistance in the context of UTIs.

## 2. Results

### 2.1. Urinalysis Findings

From a total of 2472 urine samples collected during the study period, 378 positive samples were analyzed. This study’s findings revealed that *Escherichia coli* was the predominant bacterium isolated from urinalysis, constituting 46.3% of the total cases. *Klebsiella pneumoniae* was also frequently encountered, representing 20.6% of the isolates, which highlighted these two bacteria as potential principal agents in urinary tract infections observed in the study. *Enterococcus* spp. was found in 10.1% of cases, *Pseudomonas aeruginosa* in 6.9%, and *Proteus* spp. in 5.0%, indicating a considerable bacterial diversity. In contrast, *Acinetobacter baumanii* appeared in a mere 1.9% of cases. Additionally, other Gram-negative and Gram-positive bacteria were less common, accounting for 7.1% and 2.1%, respectively, suggesting their lesser role in the urinary infections documented (Table 1).

This study conducted an analysis of urinary samples across three consecutive years. In 2020, a total of 513 samples were examined; 236 exhibited bacteriuria with less than 1000 CFU/mL, 184 displayed mixed flora, and 93 tested positive for urinary tract infection (UTI). The demographic distribution included 249 female patients, accounting for 48.5% of the sample population, and 264 male patients, representing 51.5%, yielding a male-to-female ratio (M/F) of 1.06.

During the subsequent year of 2021, 650 urinary samples were assessed; findings showed that 308 samples had bacteriuria with less than 1000 CFU/mL, 253 contained mixed flora, and 89 were confirmed positive for UTI. The cohort comprised 312 females (48%) and 338 males (52%), with an M/F ratio of 1.08.

In 2022, the number of cultures expanded to 1309 samples. Of these, 661 were identified with bacteriuria under 1000 CFU/mL, 452 had mixed flora, and 196 were positive for UTI. The participant distribution was more balanced, with 664 female patients (50.7%) and 645 male patients (49.3%), leading to a female-to-male ratio (F/M) of 1.02. The data collected over the three years provided valuable insights into the prevalence and distribution of urinary tract infections, along with the bacteriological landscape within the sampled population, as presented in Table 2.

In the year 2020, the analysis was conducted on 513 samples, with 93 identified as positive for uropathogens. Isolates included *Escherichia coli* (44 instances), *Klebsiella pneumoniae* (21 cases), *Enterococcus* spp. (8 cases), *Pseudomonas aeruginosa* (6 cases), *Proteus* spp. (3 cases), and *Acinetobacter baumanii* (2 cases), alongside other Gram-negative (7 cases) and Gram-positive bacteria (2 cases). Out of the total *Escherichia coli* strains, 11 (representing 25%) were found to produce ESBL. *Klebsiella pneumoniae* strains displayed a 52.3% positivity for ESBL, with four strains (19.04%) demonstrating extended drug resistance. Remarkably, half of the isolates belonging to *Pseudomonas aeruginosa* and *Acinetobacter baumanii* were identified as carbapenemase producers.

In 2021, out of the 650 analyzed samples, 89 tested positive. The isolates comprised *Escherichia coli* (40 cases), *Klebsiella pneumoniae* (11 cases), *Enterococcus* spp. (8 cases), *Pseudomonas aeruginosa* (14 cases), *Proteus* spp. (6 cases), *Acinetobacter baumanii* (2 cases), as well as other Gram-negative (6 cases) and Gram-positive bacteria (1 cases). Of the *Escherichia coli* strains, 32.5% (13 cases) were ESBL producers. Among the *Klebsiella pneumoniae* isolates, 18.1% were ESBL positive, with four (36.3%) being carbapenemase positive and presenting extended drug resistance. A significant 78.5% of the *Pseudomonas aeruginosa* strains and all *Acinetobacter baumanii* strains were found to be carbapenemase positive.

The analysis in 2022 encompassed 1309 samples, with 196 yielding positive results for uropathogens. Isolated strains included *Escherichia coli* (91 cases), *Klebsiella pneumoniae* (46 cases), *Enterococcus* spp. (22 cases), *Pseudomonas aeruginosa* (6 cases), *Proteus* spp. (10 cases), *Acinetobacter baumanii* (3 cases), along with additional Gram-negative (13 cases) and Gram-positive (5 cases) bacteria. *Escherichia coli* isolates producing ESBL accounted for 26.3% (24 cases). For *Klebsiella pneumoniae*, 13.04% were ESBL positive, while a striking 71.7% (31 cases) were carbapenemase positive with extensive drug resistance. Moreover, 66.6% of *Pseudomonas aeruginosa* isolates and all *Acinetobacter baumanii* isolates were carbapenemase positive. Notably, 4.54% of *Enterococcus* spp. isolates were resistant to vancomycin (VRE).

The prevalence of ESBL-positive *Escherichia coli* strains was higher in 2021—32.5%, compared to 2020—25% and 2022—26.3%. Concerning the carbapenemase-positive and extensively drug resistant strains of *Klebsiella pneumoniae*, we observed a concerning growth during the three years examined: if in 2020, we had a prevalence of 19.04%, in 2021 it grew to 36.3% and it reached 71.7% in 2022. Overall, from the 175 strains of *Escherichia coli*, 27.4% (n = 48) were ESBL producing. A total of 24.3% of the *Klebsiella pneumoniae* strains were ESBL producing, and 41 of them (52.5%) were carbapenemase positive and presented extended-drug resistance (XDR). A total of 69.2% of *Pseudomonas aeruginosa* strains and 85.7% of *Acinetobacter baumanii* strains were carbapenemase positive. Among Gram-positive bacteria, 2.6% of *Enterococcus* spp. strains were VRE, as presented in Table 3.

### 2.2. Escherichia coli

**Figure 1:**
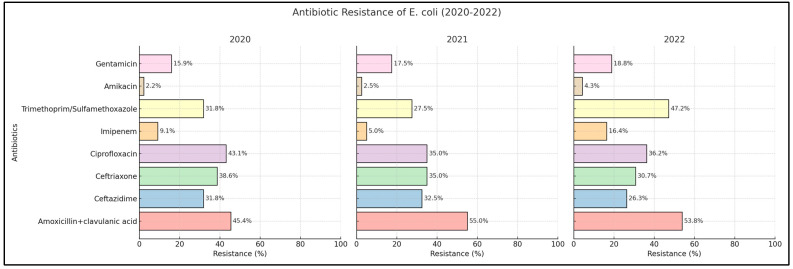
Antibiotic resistance of *Escherichia coli* across 2020 to 2022.

Our investigation revealed notable levels of resistance among *Escherichia coli* strains against amoxicillin + clavulanic acid, with resistance frequencies recorded as 45.4% in 2020, 55% in 2021, and 53.8% in 2022. The resistance rates for third generation cephalosporins displayed variations over the study period. Specifically, in 2021, the resistance percentage for ceftazidime was recorded as 32.5%, higher than the rates of 31.8% in 2020 and 26.3% in 2022. Similarly, for ceftriaxone, the maximum resistance percentage was observed in 2020 at 38.6%, followed by a decrease to 35% in 2021 and further down to 30.7% in 2022. Examination of individual urine samples indicated resistance rates to fluoroquinolones, notably ciprofloxacin, at 43.1% in 2020, followed by a decline to 35% in 2021 and 36.2% in 2022. Resistance to carbapenems, particularly imipenem, increased from 9.09% in 2020 to 16.4% in 2022. Moreover, our research findings documented a rise in resistance rates for the trimethoprim/sulfamethoxazole combination, from 31.8% in 2020 to 47.2% in 2022, as presented in Figure 1.

### 2.3. Klebsiella pneumoniae

Our findings indicate a notable escalation in the resistance of *Klebsiella pneumoniae* to amoxicillin + clavulanic acid, with frequencies reaching 57.1% in 2020, 72.7% in 2021, and 89.2% in 2022. Regarding third generation cephalosporins, the resistance percentages peaked in 2022, with 84.7% for ceftazidime compared to 57.1% in 2020 and 63.6% in 2021 and 84.7% for ceftriaxone, up from 61.9% in 2020 and 63.6% in 2021. Analysis of individual urine samples demonstrated a progressive increase in fluoroquinolone resistance rates over three years, with ciprofloxacin resistance rising from 61.9% in 2020 to 63.6% in 2021 and further to 86.9% in 2022.

**Figure 2:**
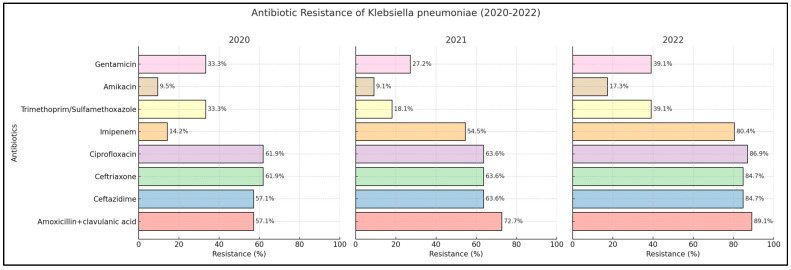
Antibiotic resistance of *Klebsiella pneumoniae* across 2020 to 2022.

The resistance to carbapenems, particularly imipenem, showed a substantial increase from 14.2% in 2020 to 80.4% in 2022. Additionally, resistance rates to the trimethoprim/sulfamethoxazole combination rose from 33.3% in 2020 to 39.1% in 2022. Among aminoglycosides, amikacin resistance increased from 9.5% in 2020 to 17.3% in 2022, while gentamicin resistance rose from 33.3% in 2020 to 39.1% in 2022, as presented in Figure 2 and Table 4.

The analysis of antibiotic resistance trends for *Escherichia coli* and *Klebsiella pneumoniae* between 2020 and 2022 revealed statistically significant findings, particularly in the increasing resistance patterns of *Klebsiella pneumoniae*. For this pathogen, resistance to amoxicillin + clavulanic acid dramatically increased from 57.1% in 2020 to 89.1% in 2022, with a *p*-value less than 0.001, indicating a strong and significant trend. Similarly, resistance to ceftazidime, ceftriaxone, and ciprofloxacin also showed significant upward trends, with final resistance rates reaching 84.7%, 84.7%, and 86.9% respectively, all with *p*-values less than 0.001. Additionally, resistance to imipenem surged from 14.2% to 80.4% (*p*-value < 0.001). For *Escherichia coli*, notable increases were observed in the resistance to imipenem from 9.09% to 16.4% and to trimethoprim/sulfamethoxazole from 31.8% to 47.2%, both achieving statistical significance with *p*-values of 0.026 and 0.009, respectively.

## 3. Discussion

This study revealed alarming trends in antimicrobial resistance among uropathogens during the COVID-19 pandemic, by analyzing urinary samples from three consecutive years. In 2020, 25% of *Escherichia coli* isolates produced ESBLs, increasing to 32.5% by 2021. Klebsiella pneumoniae showed a more severe trend, with ESBL positivity rising from 52.3% in 2020 to 71.7% in 2022, accompanied by escalating carbapenem resistance. By 2022, the resistance in *Pseudomonas aeruginosa* and *Acinetobacter baumannii* to carbapenems was also high.

The findings of our study on urinary tract infections during the COVID-19 pandemic, highlighting the predominance of *Escherichia coli* (46.3%) and *Klebsiella pneumoniae* (20.6%) as principal uropathogens, align closely with the recent literature. A Romanian study reported *Escherichia coli* in 60.95% of UTI cases and *Klebsiella species* in 17.25% of cases during the COVID-19 pandemic [33]. Similar trends in antimicrobial resistance have been observed, such as substantial resistance rates to ciprofloxacin of 65% for *Escherichia coli* and 62% for *Klebsiella pneumoniae* and a 38% resistance rate to carbapenems for *Klebsiella pneumoniae* reported in Italy [34]. These figures underscore the heightened challenge of managing drug-resistant infections, intensified by the pandemic’s impact on antibiotic prescribing practices [35], and highlight the critical need for robust antimicrobial stewardship.

*E. coli*, being the most commonly isolated uropathogen, exhibited an increase in resistance to commonly used antibiotics during the COVID-19 pandemic period, suggesting a higher adaptability and increased antibiotic use. The implications are multifaceted: there is a direct impact on patient outcomes, an increased burden on healthcare systems, and a catalyst for the need to develop alternative therapeutic strategies. Alarmingly, the resistance to carbapenems, antibiotics often reserved for multidrug-resistant infections, has risen over the study period. This increase could limit the efficacy of last-resort treatments and signals an urgent need for antibiotic stewardship programs.

*Klebsiella pneumoniae* also showed an upward trend in resistance, particularly to amoxicillin–clavulanic acid and cephalosporins. The rise in carbapenemase-producing strains over the three years is an indicator of the public health challenge posed by these resistant pathogens. The escalation of drug-resistant strains could be associated with hospital-acquired infections, where high antibiotic use is prevalent, suggesting that hospital infection control measures and antimicrobial stewardship are vital [36].

As generally observed across studies, female gender is the most affected by UTIs, as also described in the current research. Several risk factors are implicated in the heightened prevalence of UTIs, among which female gender is prominent, alongside sexual activity, diabetes mellitus, prior UTIs, genetic factors, and obesity [37]. Among premenopausal women, risk factors include frequent sexual intercourse, shifts in vaginal flora, contraceptive methods involving spermicides or diaphragms, a history of UTIs during childhood or within the family, new sexual partnerships, the absence of urination post-coitus, and suboptimal hygiene practices [38,39]. For postmenopausal women, the risk is influenced by urinary incontinence, reduced trophic hormones affecting the genitourinary tract, anterior vaginal wall prolapse, urinary catheterization, and increased postvoid residual volume [39]. 

Surveillance data are instrumental in discerning the temporal trends in antimicrobial resistance, determining whether interventions are steering towards a favorable outcome or if continued, targeted approach to antimicrobial resistance management is necessary. The management of complex UTIs hinges on the accurate identification of causative uropathogens and their susceptibility profiles to prevent the misuse of antibiotics [35,40]. Recent studies have suggested that the COVID-19 pandemic has influenced bacterial resistance patterns, likely due to the inadvertent use of antibiotics for viral infections, reinforcing the importance of local surveillance to guide effective antimicrobial stewardship and ensuring the delivery of quality healthcare in the management of UTIs [41,42,43,44].

In the current study, positive cultures were identified in only 15.3% (378 samples), diverging from higher prevalence rates reported in other studies, which ranged from 30% [40] to 70.83% [43]. Such discrepancies may be attributable to variations in demographic characteristics, local environmental factors, and differing methodologies employed across studies.

*Escherichia coli* and *Klebsiella pneumoniae* were identified as the most frequent uropathogens, comprising 46.2% (175 cases) and 20.6% (78 cases) of the isolates, respectively, aligning with findings from research conducted in other geographical regions, such as Egypt [41] and Germany [45]. The high incidence of these bacteria highlights their significance as targets for monitoring antibiotic resistance trends.

High antibiotic resistance has consequential effects, leading to increased rates of treatment failure, prolonged hospital stays, higher healthcare costs, and mortality, exacerbated by the current dearth of novel antimicrobial agents [45]. In this investigation, resistance to aminoglycosides and carbapenems was notably lower among *E. coli* isolates compared to other antibiotics, with amikacin showing a resistance rate of 4.3% in 2020 and gentamicin increasing to 18.8% in 2022. Resistance to carbapenems, such as imipenem, was registered at 16.4% in 2022. Conversely, *Klebsiella pneumoniae* exhibited escalating resistance trends across all antibiotic classes over the study period.

Given the imperative that empirical therapy for severe infections should be guided by a resistance threshold below 10% to maintain efficacy [46], our findings indicate that most resistance rates exceed this threshold, narrowing the arsenal available for managing urosepsis. Moreover, *Escherichia coli* demonstrated notably high resistance rates to amoxicillin–clavulanic acid (53.8% in 2022) and trimethoprim–sulfamethoxazole (47.2% in 2022). *Klebsiella pneumoniae* exhibited even more pronounced resistance, with over 80% resistance observed in 2022 across several antibiotic classes including penicillin + beta-lactamase inhibitors (89.1%), third-generation cephalosporins (84.7%), fluoroquinolones (86.9%), and carbapenems (80.4%). These findings underscore the impracticality of using these antibiotics as empirical therapy for UTIs due to the high likelihood of resistance, a conclusion supported by similar resistance profiles reported in other studies, such as those by Mirsoleymani et al. [47].

Extended drug resistance is characterized by resistance to at least one agent in all but two or fewer antimicrobial categories [45]. The challenge posed by XDR pathogens, particularly in Gram-negative bacteria, has been substantiated by research, including studies by Labah et al. [43], and represents a critical hurdle due to the limited treatment options available. 

The observed antibiotic resistance rates in our study are concerning, particularly in the context of potential nosocomial infections. To accurately monitor and respond to the evolving landscape of bacterial resistance, systematic surveillance of antimicrobial sensitivity and resistance over time is essential, as the current study aimed for. Preliminary evidence suggests that the COVID-19 pandemic has influenced bacterial resistance patterns, likely due to the increased use of antibiotics, which were more freely available during the pandemic [48,49]. Furthermore, consistent with the World Health Organization (WHO) guidelines, antibiotics should not be prescribed for therapeutic or prophylactic purposes in cases of mild to moderate disease without clear clinical indications, to prevent unnecessary escalation of resistance [49]. 

Our findings, reflective of the antibiotic resistance trends during the COVID-19 pandemic, reveal an increase in resistance rates by the end of 2022, a phenomenon that has been paralleled in other parts of the world. The disruption of normal healthcare operations during the pandemic may have exacerbated this issue, emphasizing the need for global cooperation in combating antibiotic resistance. Future strategies must prioritize antimicrobial resistance surveillance, adherence to infection control protocols, and investment in research for novel antimicrobials and new drug formulations, as well as treatment approaches to protect the efficacy of existing antibiotic therapies [50,51,52].

This study has several limitations, primarily due to its retrospective nature and the reliance on archived data from a single hospital, which may limit the generalizability of findings across different regions or healthcare settings. The focus on only samples testing positive for UTIs might also introduce selection bias, potentially overlooking asymptomatic or undiagnosed cases that could exhibit different resistance patterns. Furthermore, the lack of longitudinal patient data prevents tracking changes in resistance over time within individual patients, limiting our understanding of resistance development dynamics. Moreover, outpatients were not enrolled due to a lack of availability of data, potentially increasing the risk of bias for analyzing only the urine samples from admitted patients.

## 4. Materials and Methods

### 4.1. Study Design

During the period from January 2020 to December 2022, we designed a surveillance cross-sectional study that included the analysis of urine samples collected from patients admitted to the Clinical Hospital of Pulmonology and Infectious Diseases “Dr. Victor Babes” in Timisoara. All samples coming from the hospital database during the specified study period were from unique patients who had tested positive for UTIs.

Ethically, this study adhered to all applicable institutional and national guidelines for the care and use of clinical samples. Approval for this study was obtained from the hospital’s Ethics Committee of the “Dr. Victor Babes” Hospital, ensuring compliance with the ethical standards of the Declaration of Helsinki. Informed consent from individual patients was waived due to the retrospective nature of the analysis, which involved the use of existing medical records and samples collected during routine clinical care. All patient data were anonymized and de-identified prior to analysis to maintain confidentiality and protect personal information.

### 4.2. Data Collection and Definition of Variables

Data collection was performed from the hospital’s database and patients’ paper records, focusing on the urine samples. Each sample underwent urinalysis, bacterial culture, and assessment of antibacterial susceptibility. The variables defined included the type of bacteria isolated (e.g., *Escherichia coli*, *Klebsiella pneumoniae*), their prevalence as a percentage of total positive cases, and resistance patterns to various antibiotics. Demographic data such as age, gender distribution, and comorbid conditions (e.g., diabetes, hypertension) were recorded to correlate urinary tract infections with patient characteristics. Additionally, variables like the presence of bacteriuria (defined as bacteria counts <1000 CFU/mL), mixed flora, and confirmed UTI cases were analyzed annually to observe temporal shifts in bacterial populations and resistance trends, in order to interpret changes in the epidemiological landscape of urinary infections during the study period.

### 4.3. Definitions

In the current study, bacteriuria was used to refer to the presence of bacteria in the urine. Bacteriuria levels below 1000 colony-forming units per milliliter (CFU/mL) were considered indicative of an absence of significant bacterial growth and hence were not clinically significant [21]. Mixed flora classification was applied when urine cultures demonstrated the presence of multiple organisms, with at least two distinct species identified in addition to the primary organism. This condition often complicates the diagnosis and management of urinary tract infections due to the variety of potential pathogens involved [22]. UTIs were defined as infections occurring when bacteria entered the urethra and subsequently infected the urinary tract. The pathogens responsible typically originated from the skin or the rectum. For diagnostic purposes, a significant bacterial load, specifically greater than 105 CFU/mL in the urine, was considered indicative of an active infection [22].

### 4.4. Statistical Analysis

Statistical analyses for this study were conducted using SPSS Version 26.0. Descriptive statistics summarized demographic and clinical characteristics, employing means and standard deviations for continuous variables and frequencies and percentages for categorical variables. Inferential analyses, including Chi-square or Fisher’s exact tests, were used to examine differences in bacterial prevalence and resistance patterns across the study period. All tests were two-tailed, with *p*-values less than 0.05 considered statistically significant.

## 5. Conclusions

This study analyzed 2472 urine samples over the three years of the COVID-19 pandemic (2020 to 2022), identifying a significant prevalence of *Escherichia coli* and *Klebsiella pneumoniae*. A concerning escalation in antimicrobial resistance was noted, particularly for *Klebsiella pneumoniae*, which showed an increase in carbapenemase production and resistance to third-generation cephalosporins. The prevalence of ESBL-producing *Escherichia coli* also increased, highlighting the need for enhanced surveillance and strategic antibiotic stewardship to manage resistance trends effectively. The data emphasize the critical importance of continuous monitoring of antimicrobial susceptibility patterns to guide empirical treatment strategies and mitigate the impact of resistance in the clinical setting.

## Figures and Tables

**Table 1:** Background characteristics and urinalysis findings.

Variables	*n*	%
Background information		
Age (mean ± SD)	53.6	16.1
Men	120	31.7%
Women	258	68.3%
Comorbidities		
Diabetes	73	19.3%
Chronic kidney disease	31	8.2%
Hypertension	140	37.0%
Cardiovascular disease	94	24.9%
Other	22	5.8%
Bacteria		
*Escherichia coli*	175	46.3%
*Klebsiella pneumoniae*	78	20.6%
*Enterococcus* spp.	38	10.1%
*Pseudomonas aeruginosa*	26	6.9%
*Proteus* spp.	19	5.0%
*Acinetobacter baumanii*	7	1.9%
Other Gram-negative bacteria	27	7.1%
Other Gram-positive bacteria	8	2.1%
Total	378	100%

SD—standard deviation.

**Table 2:** Bacteria isolated from urinalysis.

2020			2021			2022		
Bacteriuria <1000 CFU/mL	Mixed flora	UTI	Bacteriuria <1000 CFU/mL	Mixed flora	UTI	Bacteriuria <1000 CFU/mL	Mixed flora	UTI
236	184	93	308	253	89	661	452	196
Total = 513			Total = 650			Total = 1309		

UTI—urinary tract infection; CFU—colony forming unit.

**Table 3:** Antimicrobial patterns of bacteria isolated from urinalysis.

*Escherichia coli* ESBL		*Klebsiella pneumoniae ESBL*		*Klebsiella pneumoniae carba+ XDR*		*Pseudomonas aeruginosa carba+*		*Acinetobacter baumanii carba+*		*Enterococcus* spp. *VRE*	
*n* = 48	27.4%	*n* = 19	24.3%	*n* = 41	52.5%	*n* = 18	69.2%	*n* = 6	85.7%	*n* = 1	2.6%

ESBL—extended-spectrum beta-lactamase; XDR—extended drug resistance; VRE—vancomycin resistant *Enterococcus* spp.

**Table 4:** Antibiotic resistance of *Escherichia coli* and *Klebsiella pneumoniae*.

%	*Escherichia coli*				*Klebsiella pneumoniae*			
	2020	2021	2022	*p*-Value	2020	2021	2022	*p*-Value
Amoxicillin + clavulanic acid	45.4%	55.0%	53.8%	0.334	57.1%	72.7%	89.1%	<0.001 *
Ceftazidime	31.8%	32.5%	26.3%	0.579	57.1%	63.6%	84.7%	<0.001 *
Ceftriaxone	38.6%	35.0%	30.7%	0.502	61.9%	63.6%	84.7%	<0.001 *
Ciprofloxacin	43.1%	35.0%	36.2%	0.445	61.9%	63.6%	86.9%	<0.001 *
Imipenem	9.09%	5.0%	16.4%	0.026 *	14.2%	54.5%	80.4%	<0.001 *
Trimethoprim/sulfamethoxazole	31.8%	27.5%	47.2%	0.009 *	33.3%	18.1%	39.1%	0.004 *
Amikacin	2.2%	2.5%	4.3%	0.642	9.5%	9.09%	17.3%	0.131
Gentamicin	15.9%	17.5%	18.8%	0.863	33.3%	27.2%	39.1%	0.203

*—statistically significant differences.

## Data Availability

The original contributions presented in this study are included in the article; further inquiries can be directed to the corresponding author.
